# FRAM-based causal analysis and barrier measures to mitigate dust explosions: A case study

**DOI:** 10.1371/journal.pone.0287328

**Published:** 2023-06-15

**Authors:** Meng Zhang, Lei Zhang, Xiong Cao, Baolin Li, Aitao Zhou

**Affiliations:** 1 School of Environment and safety Engineering, North University of China, Taiyuan, P. R. China; 2 School of Emergency Management and Safety Engineering, China University of Mining and Technology (Beijing), Beijing, China; Xi’an University of Science and Technology, CHINA

## Abstract

Both the number of dust explosion accidents and the resulting number of casualties have increased dramatically in recent years. To reduce this risk of dust explosions, we use the functional resonance analysis method (FRAM) to analyze the cause of the dust explosion accident at the Kunshan factory and propose barrier measures to prevent such accidents. The functional units that changed in the production system during the accident and how these functional units coupled to eventually cause the dust explosion were examined and explained. In addition, barrier measures were developed for functional units that changed during production and emergency systems defined to block the propagation of changes between functions and prevent resonance. Through case study, the identification of key functional parameters in both triggering the initial explosion and in then allowing its spread are key to define barriers to prevent a recurrence of such an event. FRAM uses system function coupling instead of traditional linear causality to explain the accident process, and develops barrier measures for changing function units, providing a novel thinking strategy and method for the analysis of accidents and their prevention.

## Introduction

A broad swath of industries generates a growing mass of explosive dust as a hazardous waste product [1, 2]. This includes the production of food and industrial minerals together with the mining, especially of coal [3–6], that has resulted in repeated dust explosion accidents [7–9]. Moreover, dust explosions typically trigger secondary explosions, expanding the range, reach and intensity of the accident and potentially causing significant loss of life and impact on value of the environment [10–12]. According to incomplete statistics, there were 67 dust explosion accidents in China from 2005 to 2020, with more than 547 deaths and more than 600 injuries [13]. It is known that fuel, oxidant, ignition source, suspension, and confinement are five essential elements for dust explosions [14]. Thus, safety measures eliminating one or more of these five essential factors are effective methods to prevent or diminish the hazard. For example, the use of metal tools, brooms, compressed air, and fans during line cleaning disperses combustible dust in potentially explosive concentrations and also causes it to settle on elevated flat surfaces [15], thus ameliorating the danger. Based on this, the concept of inherent safety is considered a proactive approach to elevate safety where hazards are removed or minimized to reduce risk. In this, four basic principles are used to attain an inherently safe environment, *viz*. measurement and designed-minimization, substitution, moderation, and simplification [16–18].

In search of an intrinsically safe environment, there have also been many attempts to model various dust explosion scenarios for both risk analysis and safety assessment [19]. These include methodologies for risk analysis of dust explosion based on Bayesian networks [20] where a diagnostic analysis is used and dust particle properties, oxygen concentrations, and safety training of staff are identified as the most critical root-cause events leading to dust explosions. Other analyses have used physics-based modeling of the progression of dust explosions [21] and the analysis of experimental data [22] to develop predictive models to assess the probability of dust explosions and the development of nomographs for assessing risk.

The prediction of both accidents and systems failures can be driven by an appropriate accident causation model [23]. Accident causation models are developed to systematically probe accidents, *a posteriori*, and are crucial in understanding the underlying reasons accidents occur and how to then improve system safety [24]. The FRAM protocol [25–28] provides a structured method to develop an overall understanding of how a complex sociotechnical system operates. FRAM can be applied by identifying functions with detailed information about how a task is completed, characterizing the variability of the functions, interpreting possible couplings of the variability, and providing suggestions to manage any unexpected variability [29]. Thus, FRAM is potentially an effective and applicable tool for the analysis of dust explosions, but there are few related applications. We use FRAM to analyze the cause of a particularly serious aluminum dust explosion accident that occurred in the Kunshan factory in Jiangsu province on August 2, 2014. We apply FRAM to examine the dynamics of the complex social-technological systems manifest in this factory, together with the nonlinear coupling relationships amongst these elements, and use this analysis to propose barrier measures.

## Background

### FRAM analysis mechanism

The functional resonance analysis method (FRAM) may be applied to explain underlying mechanisms contributing to accidents. This is based on the principle of functional resonance, which in turn stems from the concept of stochastic resonance [30]. Stochastic resonance (SR) posits that the signal-to-noise ratio (SNR) can be enhanced by adjusting the noise intensity to a certain value under the premise of regular steady-state signal input. Functional resonance refers to the case when a functional performance changes slightly and regularly, then other functions will change through a coupling connection. Other functions in the system, as the operating environment of this function, will show irregular fluctuations due to the superposition of functional changes. If the regular change of function and the irregular fluctuation of operating environment produce a coupling, the system change will exceed positive constant range, eventually leading to system failure [29, 31]. At this time, the regular fluctuation of the function is equivalent to ‘steady-state signal input’, while the irregular change of the operating environment is equivalent to ‘noise’. If the two are coupled, the fluctuation value of the system will exceed the normal limit and cause accidents.

According to the FRAM, any function in the system is not stable and will change due to various factors, but the system can automatically adjust the functional change, so that the final output of the system is normal. However, if the system functional changes and the irregular fluctuation of the operating environment are coupled to produce resonance, the change value at this time has exceeded the range that the system can automatically adjust, the system will deviate from the normal operation direction, and eventually lead to accidents.

### FRAM analysis process

**Fig 1:**
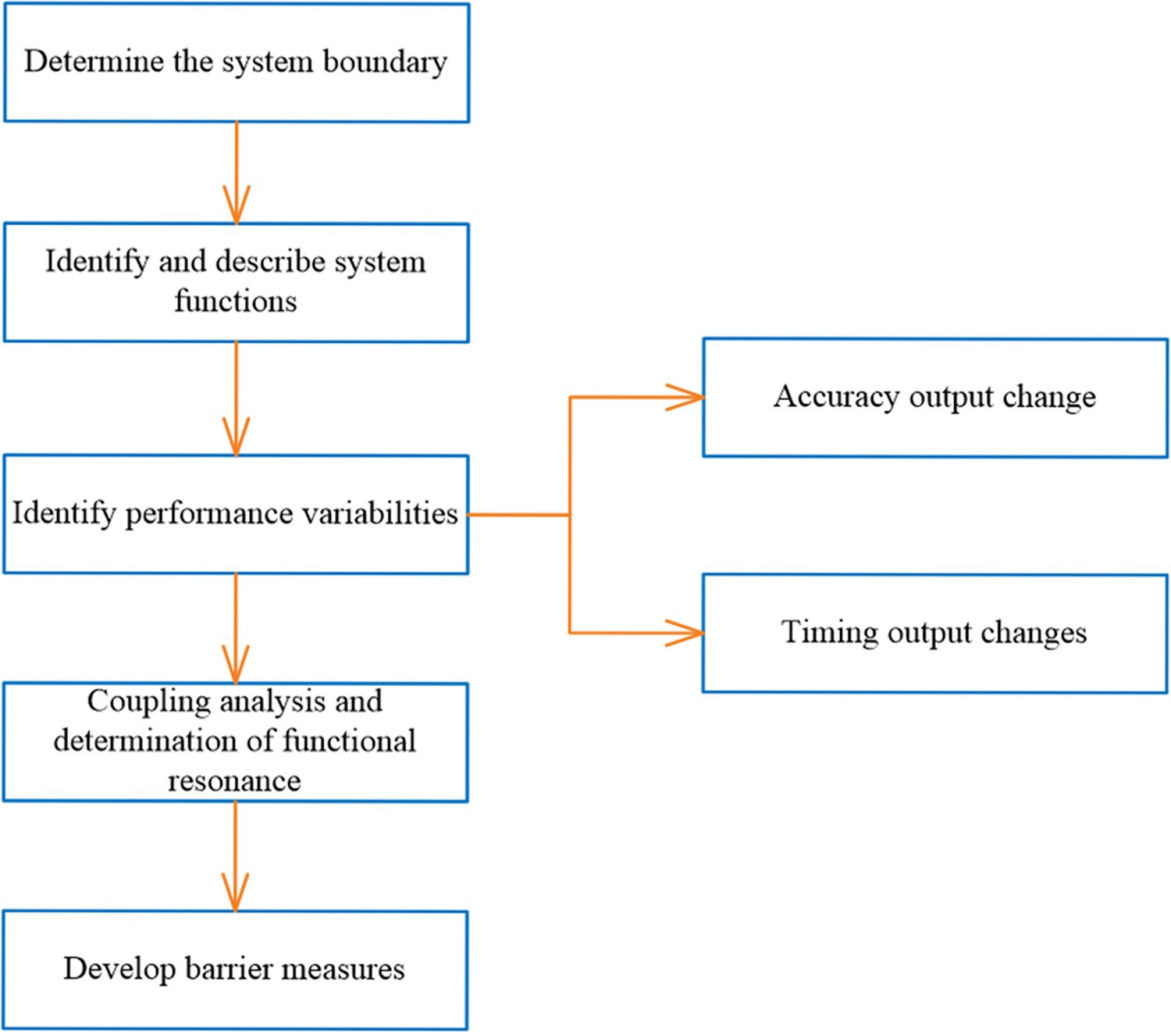
FRAM analysis process.

The process of analyzing accidents with FRAM is shown in Fig 1. The method relies on progressing through a specified sequence of steps. These are as follows:

(1) Determine the system boundary and identify the basic functions

The premise of FRAM in either risk analysis or accident case analysis is to establish a system to be analyzed. After that, the system elements should be divided into functions. The identification of basic functions is to identify a series of activities in the system.

(2) Characterize functions with parameters

**Fig 2:**
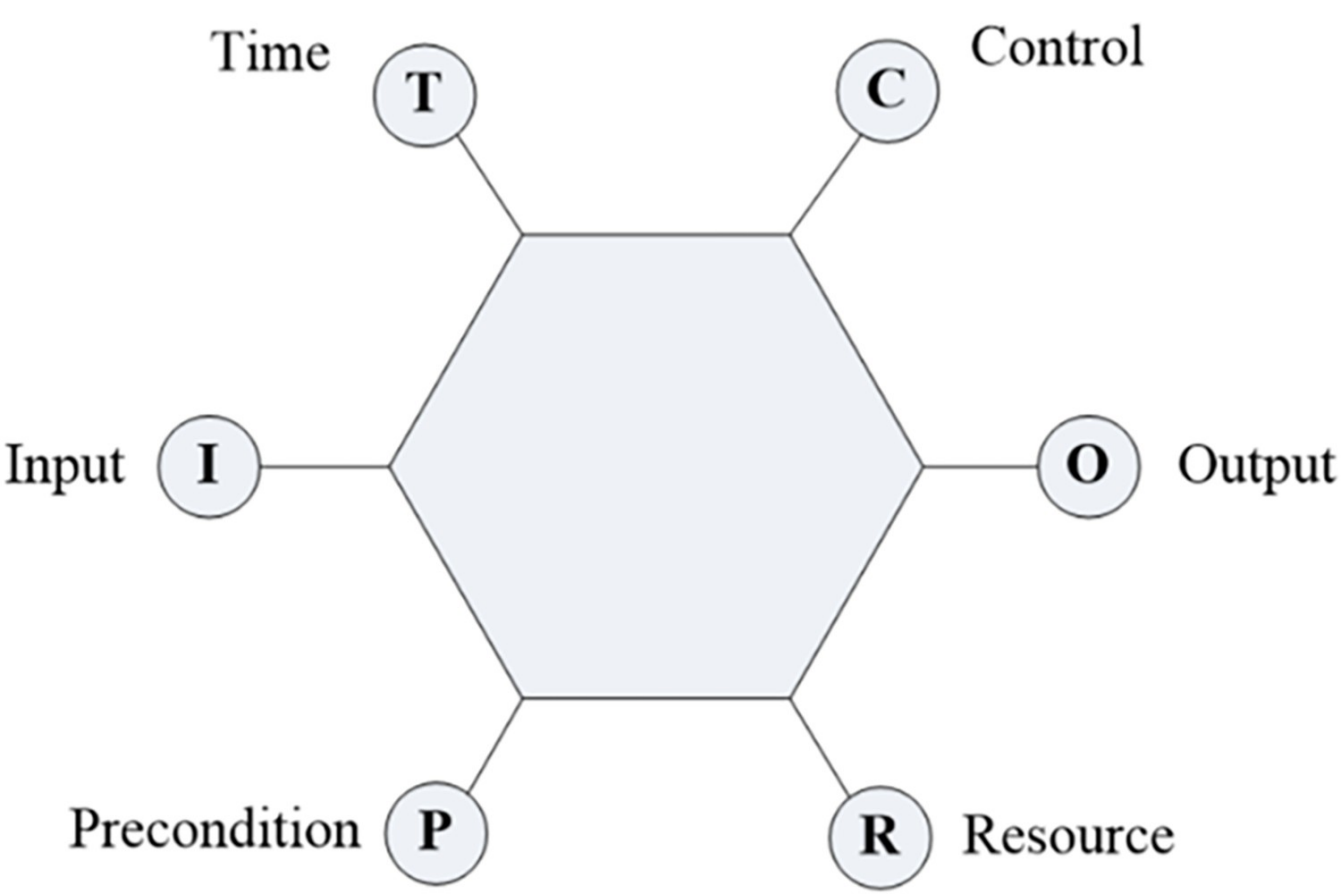
Description of system functions.

Six parameters can be used to describe the basic performance of the function. They are input, output, resource, precondition, control, and time [32] as shown in Fig 2.

The instructions for each parameter are shown in Table 1.

**Table 1:** Instructions for the parameters.

Parameter	Instruction
Input	Define functions to be dealt with
Output	Results of the functional operation
Resource	Things that need to be provided for the functional operation, such as energy, equipment, personnel, etc.
Precondition	Preconditions to be met before functional operation
Control	Constraints during the functional operation, such as procedures, laws and regulations
Time	Time conditions affecting the output results of functional operation

(3) Build a functional network diagram

The output of one function in the system can be used as other characteristic parameters except not as the output of other functions. In this way, upstream functions can communicate with downstream functions through the same parameter characteristics to establish a functional connection channel. The connection channels between all functions form a system functional network diagram.

(4) Identify performance variabilities

Functional performances are variable during operation, and may be changed due to the coupling of upstream functional performance or due to their own potential changing performance. So there are two reasons for the change:

① Coupling of upstream functions

Coupling links between functions are established through common parameters. If the upstream function changes, it may affect the operation of the downstream function through the coupling effect, so that its function will also change.

② Functional changes

The change of a function depends on its potential change performance, which can be studied from the function classification. For the implementation subject of functions, they can be divided into personnel, technology and organizational functions [33]. Different types of functions have different possibilities and reasons for change. Compared with the other two functions, personnel functions are more likely to change. The operation of technical functions is not easily affected by the external environment, and the possibility of change is lower than that of personnel and organizations.

The performance variabilities of system function can be directly reflected as the change of function output, that is, the output of function deviates from the expected goal. Function output changes are divided into accuracy output changes and timing output changes. When using FRAM for accident case analysis, you can specifically analyze which functions have changed in accuracy and timing output according to the actual situation of the accident, so as to determine the functional unit that has changed for subsequent resonance analysis; using FRAM for risk analysis, the potential changing performance of each function should be evaluated to judge the function that may have changed.

(5) Coupling analysis to determine functional resonance

Based on the identified functional performances that have changed, the failure connection between the functions is determined, and a coupling analysis of the changed functional performances and the failure connection is conducted, so as to determine how the coupling between functions cause the system resonance and eventually lead to accidents.

(6) Development of barrier measures

The goal of accident analysis is to prevent similar accidents from reoccurring. When using the FRAM to analyze accidents, barrier measures are established for functions with large changes in performance to control changes in function performance and to prevent resonance. Barrier measures are specifically divided into four categories, as shown in Table 2.

**Table 2:** Types of barrier measures.

Type	Definition	Examples
Physical barrier	To prevent accidental transfer of energy	Explosion suppression system
Functional barrier	Preconditions to be met before action	Sound and light alarm
Symbolic barrier	Indicative measures to prevent functional changes	Safety signs, warnings
Invisible barriers	Non-physical restraint barriers	Management systems, safety culture, restrictions on laws, regulations and standards

## Case study

**Fig 3:**
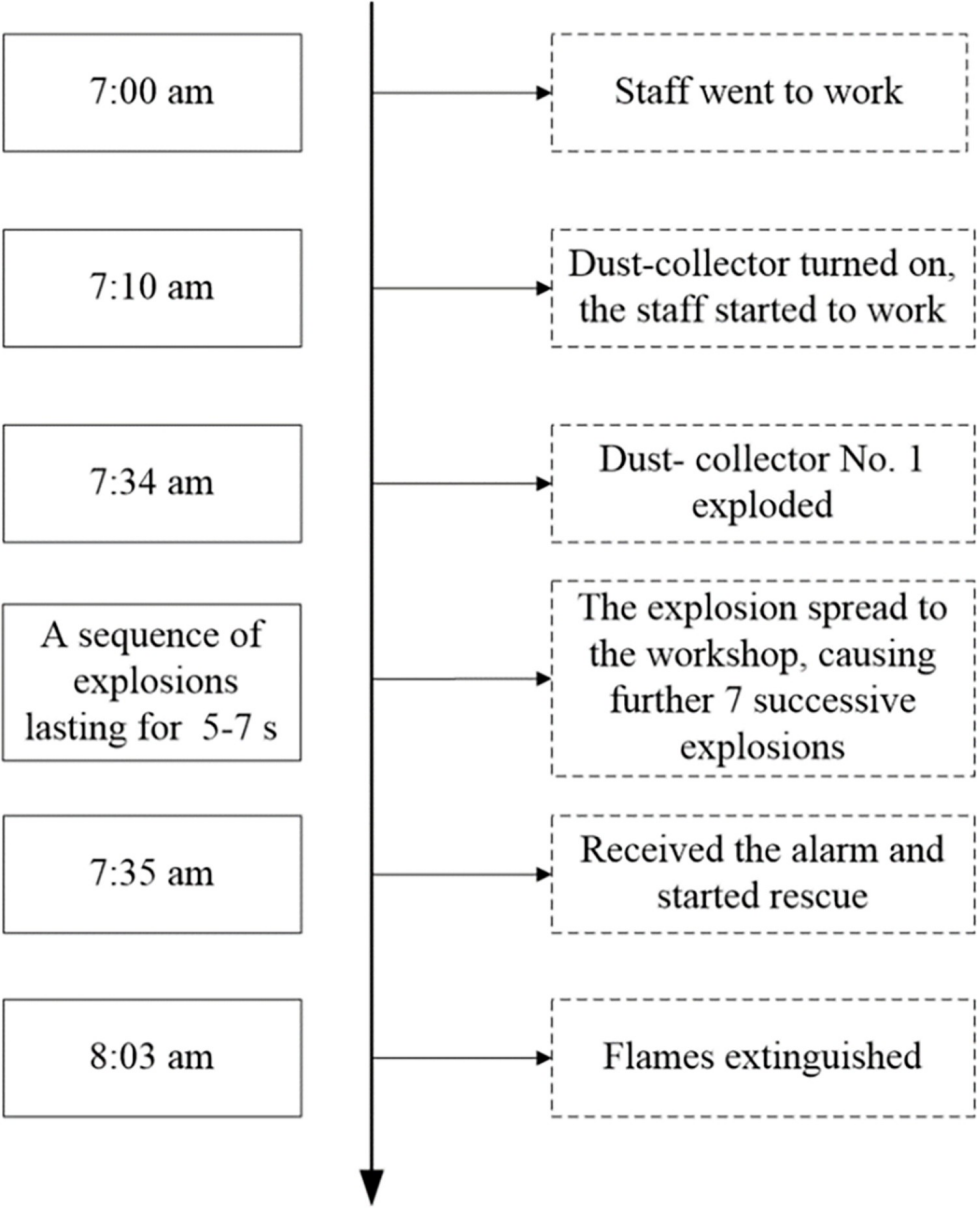
Sequence diagram of the accident.

We apply FRAM analysis to a catastrophic dust explosion in China as a case study [34, 35]. At 7:34 on August 2, 2014, a particularly serious explosion of aluminum-alloy dust occurred in the second polishing workshop (namely plant 4) of the Kunshan factory in Jiangsu province. This occurred during manual polishing of the surfaces of aluminum-alloy wheel hubs for the car industry. There were 75 immediate fatalities with another 185 injured. Subsequently, 71 of the most seriously injured also died, increased the total number of fatalities to 146. The direct economic loss was 351 million yuan (~$52M). The timeline of the accident is shown in the sequence diagram of Fig 3.

We use FRAM to study this case, analyze the causes of the accident, and propose barrier measures to avoid similar accidents.

### Identify system functions

The main production operation in the workshop where the accident occurred was manual polishing of automobile wheels to specified finish requirements. This confirms that production operation system S1 is included in the system of the accident analysis.

The identification of the basic functions in S1 is to identify a series of production activities carried out during the normal operation in the system. According to ‘Dust Explosion Protection Safety Regulations’, it is determined that the functions included to maintain the normal operation of the system S1 are: F1 assign tasks, F2 pre-job inspection, F3 dust cleaning, F4 equipment maintenance, F5 set-up of de-dusting system, F6 grinding and polishing, F7 environmental parameter monitoring and F8 on-site safety inspection. The functions are described as follows:

Before daily production, the workshop manager arranges the tasks to be completed on that day. Then, the operators check whether the production and de-dusting equipment are in good condition, ensure that there is no dust accumulation at the operation post and in the dust hood, and that the de-dusting system pipes and dust collecting bucket have been cleaned. If the requirements are not met, timely repair is applied to the damaged equipment, the deposited dust cleaned-up and the de-dusting system started after confirming that it is in good condition. The operator must ensure that the de-dusting system has been started normally before grinding and polishing is resumed.

Environmental parameters should be monitored during operation, including temperature and dust concentration. The monitored temperature refers to the temperature in the dust collection bucket and the air inlet of the dust-collector. The dust concentration is monitored through dust concentration sensors installed in the de-dusting system pipes and fan outlet. In addition, it is necessary to arrange safety management personnel or inspectors to conduct safety inspections on the work site, check whether there is dust accumulation in the workplace, whether the staff wear personal protection equipment correctly and according to the regulations, whether the workshop is equipped with dust explosion-proof electrical appliances and whether the emergency evacuation route is unblocked.

Functional changes in the production system can be obtained through environmental parameter monitoring and on-site safety inspections. However, if the monitored functional changes are not controlled and handled in time, causing functional resonance and dust explosion, the emergency system S2 can prevent the accident damage from further expanding. S2 includes functions F9 explosion suppression, explosion venting and arrestment, and F10 emergency evacuation. In the initial stage of the explosion, the explosion flame is extinguished by the explosion suppression system, so that the explosion can be effectively controlled in the initial stage. In addition, explosion venting and arrestment devices can also reduce casualties and equipment damage. At this time when the explosion is controlled, staff can evacuate through the emergency route.

**Fig 4:**
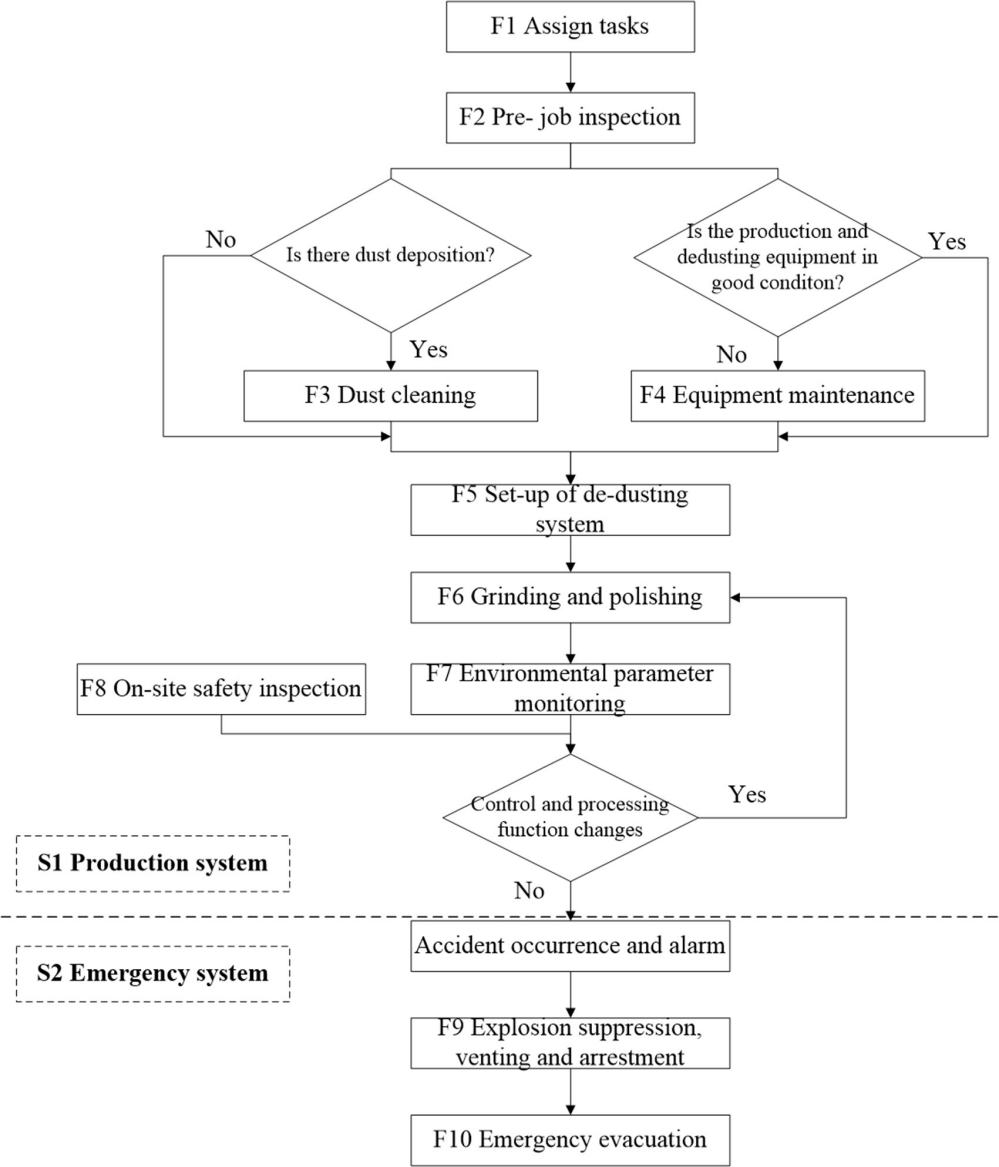
Sequence diagram of functions in S1 and S2.

The functions contained in S1 and S2, and their time sequence are shown in Fig 4.

### Describe the basic functions

Six characteristic parameters are used to describe the functions in systems S1 and S2, as shown in Table 3.

**Table 3:** Description of each function.

Function	Input	Output	Precondition	Resource	Time	Control
F1 Assign tasks	Planning of general objectives	Tasks of the day	−	−	−	−
F2 Pre-job inspection	Assigned tasks	Uncleaned dust, damaged equipment	−	Operators	Before start-up of the de-dusting system	−
F3 Dust cleaning	Uncleaned dust	The operation post, de-dusting system pipes and dust collection bucket have been cleaned	−	Operators	Before start-up of the de-dusting system	−
F4 Equipment maintenance	Damaged equipment inspected before the job	The production and de-dusting equipment is in good condition	−	Operators or professional maintenance personnel	Before start-up of the de-dusting system	Equipment maintenance management system
F5 De-dusting	Dust from polishing and grinding	The de-dusting system starts normally and the dust is separated from the air	The operation post, de-dusting system pipes and dust collection bucket have been cleaned	The de-dusting equipment is in good condition	Turn on before grinding	−
F6 Grinding and polishing	Assigned tasks	Dust from polishing and grinding	The de-dusting system starts normally; no abnormality is found in the environmental parameters and on-site safety inspection or the abnormality is controlled and processed	Operators	−	Safe operation procedures
F7 Environmental monitoring	−	The environmental parameters (temperature and concentration) are normal	−	Dust concentration sensor, temperature monitoring alarm	−	−
F8 On-site safety inspection	−	Ensure that there is no dust accumulation in the workplace, staff wear protective equipment, dust explosion-proof appliances are installed and in good condition, and emergency evacuation route is unblocked	−	Safety management personnel	−	Safety inspection management system
F9 Explosion suppression, venting and arrestment	Dust explosion	The explosion is restrained in the initial stage, overpressure is released safely, and the explosion propagation is blocked	−	Explosion suppression, venting and arrestment devices are installed in the workshop and in good condition	After dust explosion	−
F10 Emergency evacuation	Dust explosion	Staff safely evacuated outdoors	The explosion is effectively suppressed in the initial stage and the overpressure is released; the emergency evacuation route is unblocked	−	Before the explosion spreads to the work space	Emergency plan

### Build the functional network diagram

**Fig 5:**
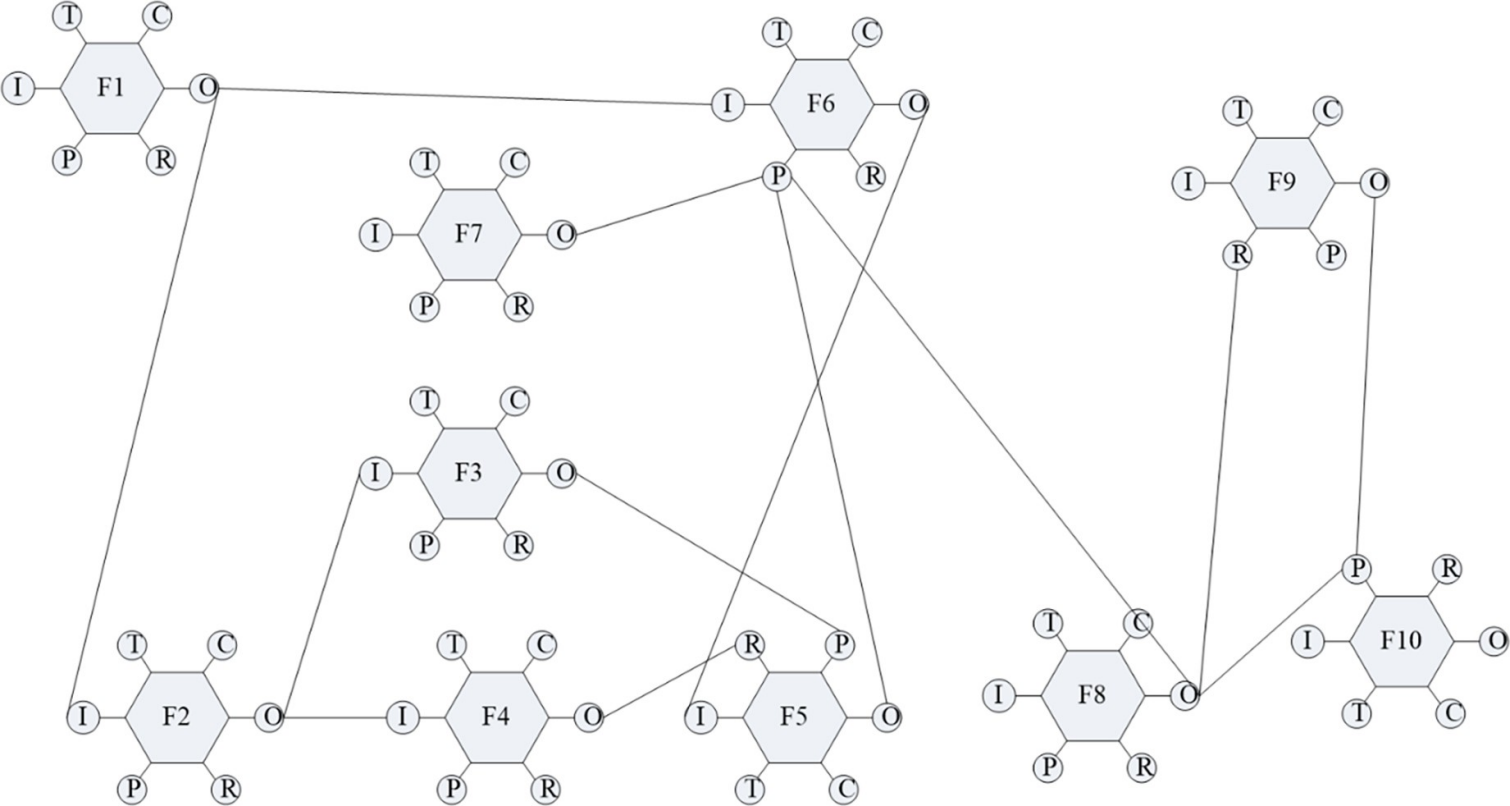
Functional network diagram.

According to the construction method, the system functional network diagram is shown in Fig 5.

### Analyze performance variabilities

According to the actual situation shown in the accident investigation report, some functions in S1 and S2 deviated from the expected target during in the actual output, causing functional resonance through coupling. The function output changes are shown in Table 4.

**Table 4:** Performance variability analysis.

Function	Nature	Accuracy output change	Timing output change
F1 Assign tasks	Organization	−	−
F2 Pre-job inspection	Personnel	Inaccuracy: The dust deposited in the de-dusting system and the rusty and worn dust collection bucket were not detected	Too late
F3 Dust cleaning	Personnel	Inaccurate: The dust in the job post, de-dusting pipes and dust collection bucket was not cleaned up in time	Too late
F4 Equipment maintenance	Personnel	Inaccurate: The rusted and worn dust collection bucket was not repaired in time	Too late
F5 De-dusting	Technology	Inaccurate: The de-dusting system did not operate properly and an explosion occurred	−
F6 Grinding and polishing	Personnel	−	Operation is not stopped immediately after the temperature and dust concentration in the dust bucket are abnormal
F7 Environmental monitoring	Technology	Inaccurate: The temperature in the dust bucket reached the ignition temperature of the aluminum dust without alarm; The dust concentration in the de-dusting pipes exceeded the limit	Too late
F8 On-site safety inspection	Organization	Inaccuracy: The employees did not wear personal protection equipment; the emergency passage was occupied; no explosion suppression system, and the explosion venting and explosion arrestment device could not work properly; dust deposition in the workplace	Too late
F9 Explosion suppression, venting and arrestment	Technology	Inaccuracy: The explosion suppression system was not installed or did not work, resulting in the flame not being extinguished at the beginning of the explosion; the dust-collector and the de-dusting pipes were not equipped with explosion arrestment and explosion venting devices, resulting in the overpressure not being released	Too late
F10 Emergency evacuation	Organization	Inaccuracy: The staff did not evacuate to the outdoor safely, causing many casualties	−

In Table 4, except for F1, the output of the other functions in systems S1 and S2 have all changed. Among them, the normal output of function F5 means that the de-dusting system is operating normally, but it is known from the accident report that the de-dusting system exploded during the operation, so function F5 is the causal unit of the accident. The output of function F10 is abnormal, causing many deaths and serious injuries, which is the unit expanding accident loss.

Among the changed functions, the functional units that changed due to their own potential performance are functions F2, F7, and F8. The output changes in these performance functions, being upstream functions, make the output of downstream functions F3, F4, F5, F6, F9 and F10 change through coupling, and finally cause resonance, leading to the dust explosion and many casualties.

### Determine functional resonance

Determining functional resonance requires to an estimation of which functional connections failed on the basis of identifying the changed functions in S1 and S2, and performing coupling analysis on the changed function units and failed connections. This is to determine how functional coupling can cause dust explosions during the operation of the de-dusting system.

(1) Failure connections

The factors influencing resonance are determined by changing functional units in S1 and S2 and the coupling relations between functions established by the same parameters. Then the functional connections leading to system failure are determined, as shown in Table 5.

**Table 5:** Functional failure connections.

Functional resonance unit	Factors affecting functional resonance	Failure connection
F2	F3 (I) Un-cleaned dust	F2 (O) —F3 (I)
F2	F4 (I) Damaged equipment inspected	F2 (O) —F4 (I)
F3	F5 (P) The operation post, de-dusting system pipes and dust collection bucket have been cleaned	F3 (O) —F5 (P)
F4	F5 (R) The de-dusting equipment is in good condition	F4 (O) —F5 (R)
F6	F5 (I) The staff shall stop the operation immediately after discovering the abnormality	F6 (O) —F5 (I)
F7	F6 (P) The environmental parameters (temperature and concentration) are normal	F7 (O) —F6 (P)
F8	F6 (P) Ensure that there is no dust accumulation in the workplace	F8 (O) —F6 (P)
F8	F9 (R) Ensure that explosion-proof appliances are installed in the workshop and in good condition	F8 (O) —F9 (R)
F8	F10 (P) Ensure that the emergency evacuation route is unblocked	F8 (O) —F10 (P)
F9	F10 (P) The explosion is restrained in the initial stage and overpressure is released safely	F9 (O) —F10 (P)

(2) Coupling analysis

**Fig 6:**
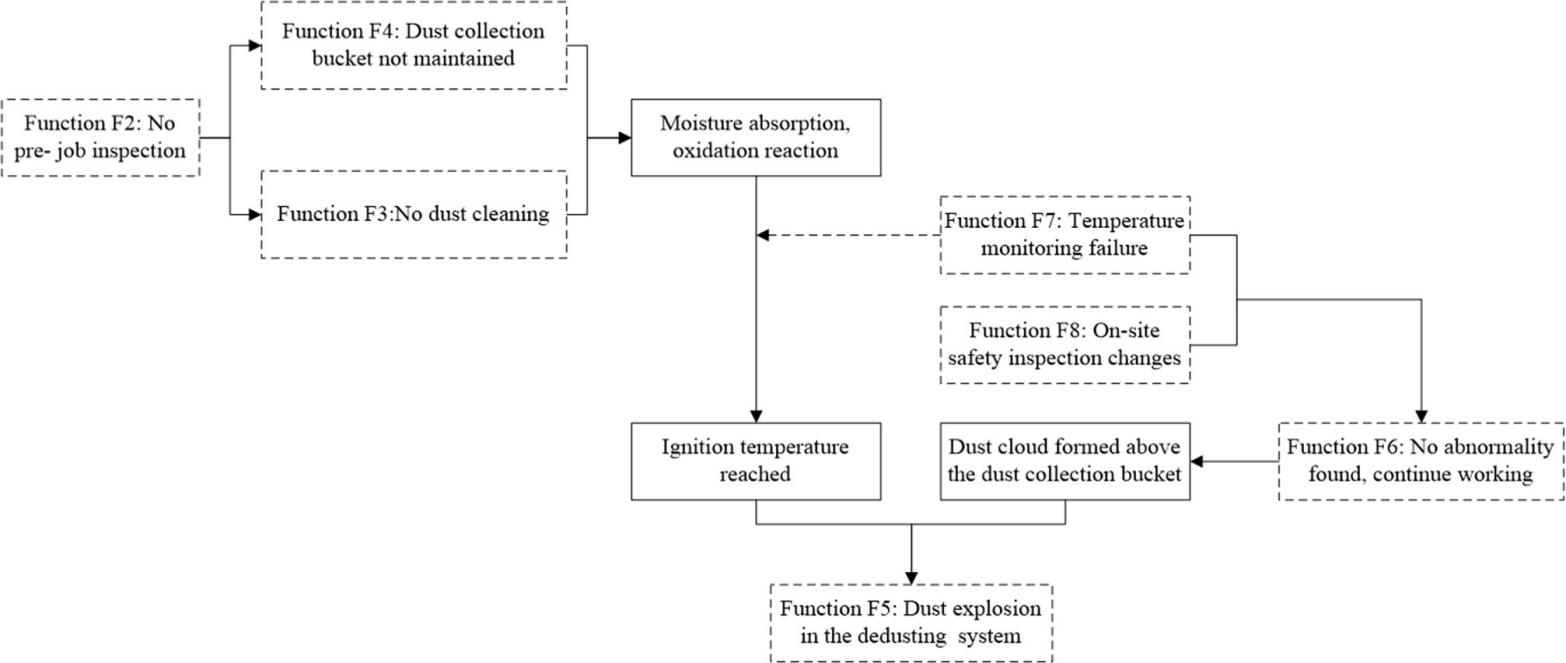
Functional coupling process of dust explosion.

The output of function F2 is the input of functions F3 and F4, and these changes cause output changes of functions F3 and F4, resulting in the rusty and worn dust collecting bucket not being repaired in time, the deposited dust in the de-dusting equipment not being cleaned in time, causing the oxidation-reduction reaction of deposited aluminum powder that is affected by moisture, and the generation of a large amount of heat. The change of F7 and F8 results in the operation system failing to timely monitor the abnormal state of temperature and dust concentration in the dust collecting bucket, culminating in reaching the ignition temperature of the aluminum powder. The staff continue to work as no abnormality is detected, and an aluminum dust cloud is formed above the dust collecting bucket. This series of functional changes are coupled and force the production system towards a critical state with the possibility of a dust explosion, which ultimately leads to the occurrence of the aluminum dust explosion. The coupling of function changes leading to the accident is shown in Fig 6.

If the emergency system S2 can act quickly after the dust explosion, the loss caused by the accident will not be increased. However, the output of function F9 in S2 has changed, which shows that the automatic fire extinguishing and explosion suppression system do not extinguish the flame in the initial stage of explosion, and that the dust-collector and de-dusting pipeline are not equipped with explosion venting and arrestment devices. This causes the explosion shock wave and combustion products to spread to the workplace along the de-dusting pipeline, further expanding the loss caused by the accident. The output of functions F8 and F9 is the premise of F10, the emergency evacuation, and their output changes affect the output of F10 through coupling, eventually producing resonance, which enlarges the loss caused by the accident.

### Develop barrier measures

To prevent accidents, barriers must be established to change functional units. For this accident, it is necessary to establish barrier measures for the changed function units F2, F3, F4, F5, F6, F7, F8, F9, and F10 to monitor and control functional changes and block the propagation path of the changes. The barrier measures developed are shown in Table 6.

**Table 6:** Barrier measures.

Functional unit	Type of barrier	Barrier measures
F2 Pre-job inspection	Symbolic barrier	Setting up prompt signs for pre-job inspection
	Invisible barrier	Develop pre-job safety inspection system
F3 Dust cleaning	Physical barrier	Strengthen the cleaning of dust deposited in the operation post and de-dusting system before the shift
F4 Equipment Maintenance	Physical barrier	Repair the damaged equipment in time
F5 De-dusting	Physical barrier	Design and install the de-dusting system in the workshop according to the specifications
	Functional barrier	Make sure that the dust cleaning is completed and the de-dusting equipment is in good condition before setting up the system
	Invisible barrier	Observe the explosion-proof safety regulations of de-dusting system
F6 Grinding and polishing	Functional barrier	Ensure that the environmental parameters and on-site safety inspection are normal before work
F7 Environmental monitoring	Physical barrier	Multi-parameter and multi-site monitoring are carried out for the workplace and de-dusting system
F8 On-site safety inspection	Physical barrier	Strengthen the safety inspection and management of the work site and timely manage hidden dangers
	Invisible barrier	Establish and comply with the hidden danger investigation and management system
F9 Explosion suppression, venting and arrestment	Physical barrier	Install dust explosion-proof appliances and ensure normal operation
	Invisible barrier	Meet the safety regulations for explosion-proof appliances in dust explosion hazardous areas
F10 Emergency evacuation	Physical barrier	Organize emergency drills regularly
	Functional barrier	Sound and light alarms are installed in the work place
	Symbolic barrier	Set the security exit logo
	Invisible barrier	Prepare emergency plan for dust explosion accident

Thus, the identification of key functional parameters in both triggering the initial explosion and in then allowing its spread are key to define barriers to prevent a recurrence of such an event.

## Conclusions

We use the FRAM protocol to analyze the cause of a dust explosion accident at the Kunshan factory in Jiangsu province on August 2, 2014. The following conclusions are drawn:

(1) The coupling of a series of function changes in the production system S1, primes the production system for a potential dust explosion that is ultimately manifest. Simultaneously, the function output of the emergency system S2 changes, producing resonance, which further expands the damage from the accident.
(2) The identification of key functional parameters in both triggering the initial explosion and in then allowing its spread are key to define barriers to prevent a recurrence of such an event. To minimize/prevent dust explosions, it is necessary to strengthen the pre-job inspection to remove dust deposits in the workplace and provide for the timely repair of damaged equipment. Any abnormalities in the system should be detected early through environmental parameter monitoring and on-site safety inspection. In addition, dust explosion-proof appliances should be installed in the plant to ensure that the emergency system can start and function normally when an accident occurs, preventing further expansion of the hazard and resulting damage.
(3) Causes of the accident and barrier measures recovered from the FRAM analysis are congruent with the conclusions of the investigation report. Both suggest mitigative measures of dust removal, equipment inspection and maintenance, the installation of dust explosion-proof appliances and the adoption of safety production management.
(4) Case analysis with FRAM identifies that a linear causal relationship between the causative factors in the traditional linear model must be replaced with a mutual coupling between functions based on the normal operation of the system functions. A new accident mechanism is proposed in FRAM which effectively accommodates the dynamics of the complex system and the nonlinear relation of elements.
